# Go for zero tolerance: Cultural values, trust, and acceptance of zero-COVID policy in two Chinese societies

**DOI:** 10.3389/fpsyg.2022.1047486

**Published:** 2022-11-22

**Authors:** Yi-Hui Christine Huang, Jun Li, Ruoheng Liu, Yinuo Liu

**Affiliations:** Department of Media and Communication, The City University of Hong Kong, Kowloon, Hong Kong SAR, China

**Keywords:** zero-COVID, collectivism, individualism, trust in government, trust in KOL, Chinese societies

## Abstract

This study seeks to explain the wide acceptance of the stringent zero-COVID policy in two Chinese societies—Mainland China (*n* = 2,184) and Taiwan (*n* = 1,128)—from perspectives of cultural values and trust. By employing the efficacy mechanism, this study identifies significant indirect effects of trust in government and key opinion leaders (KOL) on people’s policy acceptance in both societies. Namely, people who interpret the pandemic as a collectivist issue and who trust in government will be more accepting of the zero-COVID policy, whereas those who framed the pandemic as an individual issue tend to refuse the policy. Trust in government and KOLs foster these direct relationships, but trust in government functions as a more important mediator in both societies. The different contexts of the two Chinese societies make the difference when shaping these relationships. These findings provide practical considerations for governmental agencies and public institutions that promote the acceptance of the zero-COVID policy during the pandemic.

## Introduction

Starting from the end of 2019, COVID-19 has infected more than 617.6 million people worldwide and caused over 6.5 million deaths as of October 2022 (WHO, 2022). This long-lasting pandemic has sprawled and wreaked havoc on the economy as well as daily lives worldwide. It has been estimated that due to the disease, the global economy shrank by 4.4% in 2020 alone (BBC, 2021), with 6.5% of the global population out of work (The United Nations, 2021). To recover from the damage caused by the pandemic and get back to the pre-pandemic routines, countries including Denmark, South Korea, Thailand, the United Kingdom, and the United States have suggested that these countries must “live with the covid” soon (CNN, 2021; NPR, 2021; BBC, 2022), which means lifting stringent public health interventions such as social distancing and masking, deemphasizing testing, and treating the disease the same as other illnesses like influenza (Hartman, 2021). There are still countries or regions sticking with the “zero-COVID” strategy, and Mainland China as well as Taiwan are two representatives. Contrary to the “living with the covid,” the “zero-COVID” strategy utilizes rigid preventive measures including mass lockdowns, mandated testing, international travel bans, and mandatory quarantines to crush any hint of an outbreak (The Economist, 2022). From the beginning of the pandemic in December 2019, both Mainland China and Taiwan adopted this “zero-COVID” strategy. As the place where the first case of COVID-19 was discovered, Mainland China has remained vigilant about infections, and has no plan to abandon this strategy. Taiwan maintained strict intervention against the disease throughout the period of this study (December 2021). Compared with protests against the lockdowns in the West (e.g., in Germany, the Netherlands, and Austria), these two regions have endured tightened public health controls almost without public resistance. *What could explain the overall acceptance of the “zero-COVID” strategy in these two Chinese societies?*

The “individualism–collectivism” (Chen et al., 2015) dimension of cultural values has been long considered one explanation for people’s attitudes toward interventionist policies from the state. Intuitively, collectivism has been associated with more obedience and tolerance toward interventionist policies, while individualism reduces people’s preference for state intervention (Nikolaev et al., 2017; Chen et al., 2021; Kemmelmeier and Jami, 2021). Under the context of the pandemic, collectivist values have been proven to increase people’s compliance with preventive measures such as social distancing, while individualist values work in the opposite way (Wang, 2021). Specifically, a recent study on Chinese university students has demonstrated the significant effect of individual-level cultural orientations (i.e., collectivism–individualism) on people’s public health policy compliance during the COVID period (Xiao, 2021).

However, what factors give rise to such a division is still under discussion. The internal factors of “I-C” dichotomy, which is argued by Hofstede et al. (2005) as well as Pitlik and Rode (2017), may be inherent cultural traits and beliefs that lead to differences in policy acceptance (Otto et al., 2020; Atalay and Solmazer, 2021; Lyu et al., 2022). Apart from internal factors, trust has been found to play a siginificant role in many behavioral outcomes (Huang et al., 2021; Dai et al., 2022). Thus, we argue that people’s trust in government and key opinion leaders (i.e., KOLs), subjected to influences of self-efficacy and cultural values, may also shape their acceptance of zero-COVID policy. The interactions between these social actors and values of “I-C” illuminate how cultural values influence people’s policy acceptance and may generate more practical implications for practitioners in terms of mobilizing support for a given policy.

We hereby present three research questions to examine the relationship among cultural values, policy attitudes, and the role of trust in government and media: (1)*: How will cultural values, including collectivist and individualist values, predict the acceptance of the “zero-COVID” strategy in Mainland China and Taiwan?* (2)*: How will people’s trust in government and KOLs mediate the association between cultural values and the “zero-COVID” strategy?* and (3) *To what extent does the effect of cultural values on people’s policy acceptance differ in Mainland China and Taiwan?* Moreover, the research aims to contribute to the current studies on cultural values and policy acceptance by introducing the effect of self-efficacy on people’s trust and examining the interplay between cultural values and trust in policy attitudes. Policy recommendations derived from the analysis will work as the guidelines for campaigns on not only public health measures against the pandemic, but other policies as well.

### Individualism–collectivism dichotomy

Culture makes the public institutions that guide people’s behaviors. As the pandemic threatens the stability of countries around the world, numerous studies have been devoted to how different cultural dimensions—including the individualism–collectivism, uncertainty avoidance, power distance, and masculinity-femininity factors identified by Hofstede (1983)—have managed to shape people’s compliance with COVID-19 preventive measures, such as social distancing, vaccination, and self-reporting of infection (Huynh, 2020; Travaglino and Moon, 2021; Voegel and Wachsman, 2022; Lu, 2022). The dichotomy of individualism vs. collectivism has been cited as an explanation for people’s acceptance of interventionist policies before and during the pandemic (Chen et al., 2015; Travaglino and Moon, 2021). Specifically, according to Hofstede et al. (2005), “collectivism” is described as a kind of cultural value that integrates people into “strong, cohesive in-groups, often extended families that continue protecting them in exchange for unquestioning loyalty and oppose other in-groups.” In addition, studies emphasized the core principle of collectivism (e.g., the set of cultural values that make people value group interests more than individual interests; Wang, 2021; Lyu et al., 2022).

In studies related to the political culture in East Asia, “collectivism” is considered one of the major features that shapes the political institution. Shi (2014) specified several political cultural traits in Greater China, and traits like allocentric self-interest, conflict avoidance, and hierarchical orientation toward authority share common features with “vertical collectivism” (Rokeach, 1973; Fiske, 1992; Triandis and Gelfand, 1998). By this token, collectivism plays a significant role in understanding the policy attitudes of people in Greater China. Moreover, despite spending decades in diverse political and economic systems, people in China and Taiwan share similarities in cultural orientation (Head and Sorensen, 1993; Ferle et al., 2008; Bedford et al., 2021) and collectivist values (Cheung and Chow, 1999).

As for individualism, it refers to a society that is bound with loose interpersonal ties in which every member is expected to fend for oneself and his/her immediate family (Hofstede et al., 2005). The origin of this idea dates back to the Age of Enlightenment, during which the concept of individualism in philosophy involved the maximization of individual welfare and freedom (Lukes, 1971). According to Triandis et al. (1993), individualists consist of four features: (1) loosely linked persons in individual terms independent of collectives; (2) driven by own preferences, interests, and rights; (3) prioritizing the rational analyses of the benefits and drawbacks when interacting with others; and (4) individual goals outweighing collective goals, detached from their in-group members. Confrontation and competition are often ready to erupt among individualists (Triandis, 1995).

Despite the fact that both Mainland China and Taiwan are embedded in the relatively collectivist culture, collectivism is not the only element in these societies (Huang et al., 2018); individualism also plays a significant role at both the individual and societal levels (Sima and Pugsley, 2010). Individualist elements were found to be increasingly important in Chinese people’s evaluation of their personal pleasure and life satisfaction (Steele and Lynch, 2013). Particularly, Bao et al. (2022) revealed that, during the past several decades in China, individualism has grown to be acknowledged and linked with various perspectives of life (e.g., income and wealth). Moreover, Pelham et al. (2022) identified the existence of cultural shifts from collectivism to individualism in many societies, which they attribute to the factors such as the rise of national wealth and urbanization (Huggins and Debies-Carl, 2015). Empirical studies have also lent credence to this cultural shift in China (Cao, 2009). Using algorisms, Hamamura et al. (2021) analyzed 50-year printed texts in China to reveal the cultural shift and rising individualism. Against this backdrop, it is important to analyze the effect of individualism on individuals’ acceptance of policies in a comparatively collectivist culture.

### Individualism–collectivism and acceptance of interventionist policies: Employing issue interpretation as proxies

Due to differences in cultural traits, individualism has been associated with a less interventionist institution that respects the rights of others, rule of law, and is conducive to market capitalism (Tabellini, 2010). By contrast, collectivism is related to a hierarchical and orderly society that nourishes more government interventions and even authoritarianism (Kemmelmeier et al., 2003).

Popular explanations for the effect of I-C dimension and preferences over government interventions usually deal with cultural traits associated with individualism and collectivism, such as self-direction and self-determination (Pitlik and Rode, 2017). According to Pitlik and Rode (2017)’s theory, individualists find it hard to comply with interventionist policies due to high levels of both self-determination and self-direction, while collectivists are the other way around. Similar patterns are also found in existing empirical studies on preventive measures. For example, Maaravi et al. (2021) found that people ranking high in collectivist values are more likely to adhere to health guidelines issued by the government. The same conclusions are also found in studies of Wang (2021) and Bok et al. (2021), where people who have more collectivist beliefs are more willing to comply with preventive mandates.

Conversely, individualist values have been regarded as barriers to people’s acceptance of interventions from the government. The analysis from Chen et al. (2021) shows that in areas where individualistic culture was more prevalent (e.g., the United States), people were less likely to follow the lockdown regulations. Likewise, Yu et al. (2021) demonstrated in an empirical study conducted in China that stronger beliefs in individualism are associated with vaccine resistance among Chinese.

**Figure 1:**
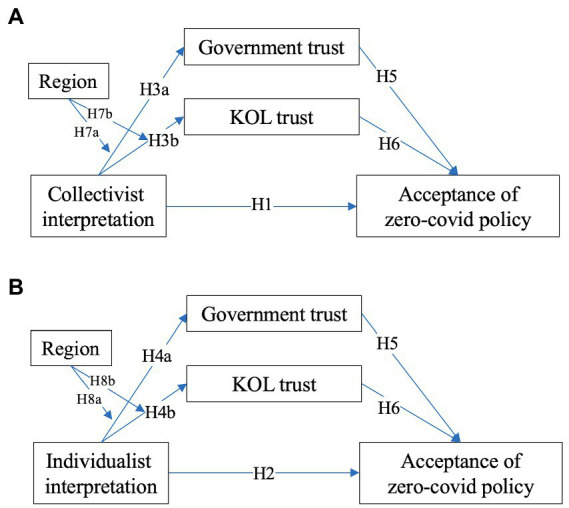
The proposed model of this study.

We hereby consider the “zero-COVID policy” a form of preventive mandates in the Chinese context since governments in Chinese societies (e.g., Mainland China and Taiwan) have insisted on the zero-COVID policy to combat the COVID. Specifically, we used respondents’ *issue interpretation of pandemic* as proxies for their values. Our use of this approach draws on various works. Smeekes and Verkuyten (2013) wrote that collectivists, whose sense of existence as well as personal identity hinges on group membership and social identity, are more motivated to defend the group interests when facing existential risks. Such collectivist beliefs can shape people’s interpretation of an issue (Travaglino and Moon, 2021) and a collective disaster framing that highlights social obligation is more palatable to them (Oyserman et al., 1998). However, individualists, whose sense of existence and personal identity are determined by personal rather than group factors (Smeekes and Verkuyten, 2013), would be more likely to accept a more individual-oriented perspective of the pandemic after evaluating the trade-offs between collective actions and their own self-determination (Travaglino and Moon, 2021). In essence, then, we have employed interpretation of pandemic as a means to discern people’s different foci on how they develop a sense of continued existence over time and space (from which they derive personal identity). Therefore, based on the existing studies reviewed, we propose the following hypotheses (Figure 1):

*H1:* People’s collectivist issue interpretation of the pandemic is positively associated with their acceptance of the zero-COVID policy.

*H2:* People’s individualist issue interpretation of the pandemic is negatively associated with their acceptance of the zero-COVID policy.

### Cultural values, political trust, and social trust: The self-efficacy perspective

Beliefs and attitudes, however, can also be shaped by external factors such as other social actors instead of being solely determined by individuals’ cultural values. Trust is another essential construct that may affect individuals’ attitudes during a certain crisis. Defined as the willingness to take risks (Johnson-George and Swap, 1982), trust has frequently been adopted to explain individuals’ decision-making processes when encountering a crisis (e.g., pandemic crisis, Algan et al., 2021; financial crisis, Earle, 2009). Cultural values have been found to affect trust in many empirical studies (e.g., Shi, 2001; Hogler et al., 2013; Xu, 2020). The underlying mechanism behind cultural values and people’s trust in certain subjects lies in their perception of self-efficacy. According to Rogers (1975), self-efficacy is people’s ability to perform a specific response. People’s self-efficacy perception will influence their decision-making, aspirations, problem-solving, etc. (Bandura, 1991). When they perceive a low level of self-efficacy in a crisis scenario, people will put more trust in other entities (e.g., government and other institutions; Rogers, 1975; Armaş et al., 2017). On the contrary, higher levels of self-efficacy assume the view of the self as competent and are assumed to be correlated with lower trust in other entities (Gecas, 1989).

Bandura (1986) suggested that self-efficacy can be socially constructed. Numerous studies have shown that individualism as a cultural value is associated with higher self-efficacy, while collectivism is associated with lower self-efficacy (Oettingen, 1995; Xie et al., 2006; Choi and Kim, 2019). Motivated by different levels of self-efficacy, individualists and collectivists invest in different types of trust relations. Due to their emphasis on higher self-reliance and self-refinement as two key components of self-efficacy (Xie et al., 2006), individualists demonstrate less trust in the government (Putnam, 2000); on the other hand, they may have higher levels of social trust because they are more autonomous and seemingly liberated from social bonds, which leads to their higher trust in each other (Realo et al., 2008). In contrast, influenced by in-group solidarity and respect for authority figures, collectivists are more likely to have higher levels of trust in political institutions (Jakubanecs et al., 2018). However, there are also studies showing that collectivism is positively associated with higher levels of social trust due to the benevolence toward others and interdependence underlying collectivist cultures (Shin and Park, 2004).

Against this backdrop, the current study will further focus on trust in government (to manifest political trust) and trust in key opinion leaders (to manifest social trust).

*Trust in the government,* as a key component of political trust, has been emphasized to explain individuals’ behaviors in response to a huge, worldwide crisis (see Blind, 2007 for a review). During a pandemic, the government functions as the headquarters of a nation. Given the role of government and the emotional value attached to nations, individuals’ trust in government is required as an in-group trust rather than relying on other types of out-group trust; citizens will rely on the government to protect them (Klein and Robison, 2020; Stevens and Haines, 2020) and comply with preventive measures based on their trust in the government (Lyu et al., 2022).

Trust *in key opinion leaders* (*KOL*) is our major measurement for social trust, as opinion leaders are wielding growing power in the public sphere under the era of Web 2.0. Opinion leaders are people with a substantial level of influence within their network and are capable of influencing others’ opinions (Shah and Scheufele, 2006; Parau et al., 2017).

Trust in KOLs can also be considered social trust because trust is developed through interpersonal communications between KOLs and members within a community through knowledge and resource sharing (Liu et al., 2019). As they function as a mediator between the media and the public, opinion leaders reinforce the acceptance of certain opinions in the public. During a pandemic, people will also rely on opinion leaders to obtain support. Due to the relationship among political trust, social trust, and the “I-C” dimension, we hypothesized (Figure 1):

*H3:* People’s collectivist issue interpretation positively predicts (a) their trust in the government and (b) their trust in KOLs.

*H4:* People’s individualist issue interpretation negatively predicts (a) their trust in the government, but positively predicts (b) their trust in KOLs.

### Trust and policy acceptance

Both *political trust* (measured by trust in government) and *social trust* (measured by trust in KOLs) can be correlated with policy acceptance. Political trust is essential in determining people’s political behaviors such as political participation (Huang et al., 2017) and policy acceptance (Rubin et al., 2009). In the realm of public health management, trust in public institutions has been associated with people’s acceptance of authorities’ recommendation (Prati et al., 2011; Travaglino and Moon, 2021; Liu et al., 2022; Wang et al., 2022). In this vein, a positive relationship between political trust and the acceptance of the zero-COVID policy can be assumed.

The relationship between social trust and policy acceptance is evidenced by illustration of links of Putnam (1993)’ among public participation, social capital, and the implementation of policies. However, in the case of pandemic, the relationship between social trust (represented by trust in KOLs in the article) and policy acceptance remains under debate (Jäckle et al., 2022). Kelly et al. (1991) indicated that the intervention with key opinion leaders would reduce HIV risk behaviors in the general population. Similarly, Nisbet and Kotcher (2009) examined the influence of opinion leader campaigns on people’s attitudes toward climate change. On the other hand, opposition from the opinion leadership results in public resistance against the policy, especially when the policy is at odds with personal judgment and well-being. In China, the public resistance against the Chinese government’s indifference on the death of Dr. Li Wenliang, a whistleblower of COVID-19, is another example of KOL-led outcry on policy issues (Tsai, 2021). As the zero-COVID policy is controversial due to its stringent intervention in people’s life routine, we propose treating opinion leaders as potential opponents of the policy because higher trust in opinion leaders may lead to lower acceptance of the policy. Given the relationship between trust and policy acceptance, we hypothesized (Figure 1):

*H5:* Trust in the government will positively predict people’s acceptance of zero-COVID policy.

*H6:* Trust in the KOLs will negatively predict people's acceptance of zero-COVID policy.

### The moderating role of regions

Additionally, we take into consideration contextual factors of two societies as possible moderators that affect the path connecting people’s cultural values with their trust. Mainland China and Taiwan share several common features in terms of political cultures, including similar beliefs in allocative self-interests, the avoidance of conflicts, and a hierarchical relationship with the authority (Shi, 2014). These similarities control the influence of cultural values between both regions, while differences in political and media environments provide the necessary comparison for how external factors influence the effect of cultural values on the policy acceptance.

Mainland China and Taiwan differ in their political system, traditionalist inclination, and preventive measures against the COVID. The political systems in Mainland China and Taiwan are distinct; since 1949, the Chinese Communist Party has ruled the mainland, but Taiwan’s political system has seen considerable changes in the past decades (Huang et al., 2016). Moreover, the level of traditionalist inclination in these two societies differs. Wong et al. (2011) suggested that a higher level of traditionalist orientation appeared in societies with a long history, such as Mainland China, and economic development does not always imply a decline in traditionalism. Furthermore, although complying with the zero-COVID policy, the two regions present certain distinctions in their strictness of preventive policies. Mainland China sticks to the rigorous zero-COVID policy, enacting lockdown measures and tightened controls in high-risk areas, while Taiwan does not plan to implement the lockdown policies as stringently as Mainland (South China Morning Post, 2022).

Given the possible distinctions between the two Chinese societies, we hypothesized a moderating role of the region on the relationship between cultural values and people’s trust in government and KOLs, as follows (Figure 1):

*H7:* Region moderates the relationships between people’s collectivist issue interpretation and (a) their trust in the government and (b) their trust in KOLs.

*H8:* Region moderates the relationship between people’s individualist issue interpretation and (a) their trust in the government and (b) their trust in KOLs.

## Materials and methods

### Participants and procedure

From November to December 2021, online surveys were conducted in Mainland China and Taiwan with participant recruitment overseen by independent agencies *Rakuten Insights* in Mainland China and *Chungliu Education Foundation* in Taiwan. Based on the 2010 Mainland population census, a mix of Probabilities Proportional to Size (PPS) sampling and quota sampling with gender and age was used in Mainland China (November to December 2021). The PPS and quota sampling were adopted for two reasons. First, as mentioned by Zhang et al. (2020), quota sampling enables researchers to target typical subpopulations with good control over the recruitment process. Second, they demonstrate the representativeness of the sample (Wartberg et al., 2014) in fixed targets concerning age and gender, and other demographic features. In summary, we adopted a combination of PPS sampling and quota sampling to ensure the samples in Mainland China were sufficiently representative with respect to the demographic characteristics (i.e., age, gender, and residence areas). In Taiwan, we utilized Random Digit Dialing (RDD) sampling based on the Taiwan 2020 household registration database (December 2021). All participants in these two regions were asked to complete an online survey on QuestionPro (in Mainland China) and SurveyCake (in Taiwan).

A total of 3,312 valid responses were received (*n_mainland_* = 2,184, *n_Taiwan_* = 1,128). Pretests with 10 respondents in each region were completed to confirm the questionnaire’s validity and reliability. Back-translation was accomplished to ensure that the original English questions were appropriately delivered (Brislin, 1970).

### Demographics of the sample

Demographic profiles (i.e., gender, age, and education) of the samples in the two societies were presented in Table 1. Gender proportion in Taiwan was close to 1:1, whereas the number of male respondents was slightly higher than females in Mainland China (1:0.808). Around 41.1% of respondents in Taiwan were over the age of 40 while only 18.1% of participants in Mainland China were in that age group.

**Table 1:** Descriptive results in two Chinese societies.

	Mainland	Taiwan
Gender (male: female)	1:0.808	1:1.055
Age (over 40)	18.1%	41.1%
Monthly household income (Mean)	CNY 15,001–25,000 (USD 2,356–3,927)	TWD 60,001–70,000 (USD 2,106–2,457)
Education (at least B.A.)	60.4%	87.5%
Top residential areas	Guangdong Province (15.2%) Shandong Province (8.3%) Jiangsu Province (6.4%)	New Taipei City (24.3%) Taipei City (12.8%)

The average income of participants in Mainland China was USD 2,356–3,927, while those in Taiwan reported a lower average income of USD 2,106–2,457. Participants in Taiwan received a higher level of education with 87.5% having at least a bachelor’s degree, compared with 60.4% in Mainland.

Participants were selected from 26 provinces or municipalities across the residential regions of Mainland China. Guangdong Province (15.2%), Shandong Province (8.3%), and Jiangsu Province (6.4%) were the top three provinces with the most participation. Respondents were recruited from 22 cities or counties in Taiwan, with New Taipei City (24.3%) and Taipei City (12.8%) ranking first and second.

### Measures

As mentioned in the Literature Review, we used issue interpretation as proxies for respondents’ I-C orientation. Detailed measurements are described as follows.

*Collectivist issue interpretation of pandemic*, as an indicator for people’s orientation toward collectivist values, was measured with three question items on a seven-point Likert scale (1 = strongly disagree to 7 = strongly agree). The question items included the “COVID-19 pandemic is mainly a social/group/national issue.” The responses were then averaged to create *collectivist issue interpretation of pandemic* (M = 5.33, SD = 1.03, Cronbach’s α = 0.78).

*Individualist issue interpretation of pandemic,* as an indicator of people’s orientations toward individualist values, was measured with two question items on a seven-point Likert scale (1 = strongly disagree to 7 = strongly agree), including the “COVID-19 pandemic is mainly a privacy/personal issue.” The responses were then averaged to create *individualist issue interpretation of pandemic* (M = 4.16, SD = 1.40, Cronbach’s α = 0.74).

*Trust in government* (*KOLs*) was measured on a seven-point Likert scale (1 = strongly distrust to 7 = strongly trust) based on previous empirical studies (Han and Windsor, 2011; Alotaibi et al., 2019). The question items in Mainland China included trust in experts, key opinion leaders on the Internet (i.e., WeChat official accounts/Weibo in Mainland and the Internet in Taiwan), and opinion leaders from TV/newspaper (M = 4.66, SD = 1.11, Cronbach’s α = 0.77).

*Trust in government* was measured on a seven-point Likert scale (1 = strongly distrust to 7 = strongly trust) based on previous studies (Cook and Gronke, 2005; Uslaner and Brown, 2005). The question items in Mainland China included trust in the central government, local government, and Centers for Disease Control and Prevention. Items in Taiwan included trust in central government, county/city government, Ministry of Health and Welfare, Centers for Disease Control and Prevention, and County/City Public Health Bureau (M = 5.36, SD = 1.23, Cronbach’s α = 0.90).

*Acceptance of the zero-COVID policy* was adapted from scale of Courneya and Bobick (2000), measured by two subgroups: attitudes toward the zero-COVID policy and attitudes toward the living with covid strategy. Under each subgroup, participants were asked “I agree with the zero covid/living with covid strategy,” “This zero covid/living with covid strategy is effective,” “This zero covid/living with covid strategy is beneficial,” and “This zero covid/living with covid strategy is wise.” Responses to the questions were recorded on a seven-point Likert scale (1 = strongly disagree to 7 = strongly agree). Answers for both subgroups were averaged first. The acceptance rates of the zero-COVID policy were computed with the average attitudes toward the zero-COVID policy deducting average attitudes toward the living with covid strategy (M = 0.92, SD = 2.06, zero-COVID strategy Cronbach’s α = 0.94, Living with the covid strategy Cronbach’s α = 0.97).

*Region* was considered as a categorical variable in which Mainland China was coded as 1 and Taiwan was coded as 0.

*Demographics*, including participants’ age, gender, education, monthly household income, and with/without children, were included as control variables.

## Results

We first used SPSS to conduct parametric correlation analyses of the study variables in two databases (Table 2) and discovered that the majority of the study variables, including collectivist issue interpretation of pandemic, individualist issue interpretation of pandemic, trust in government, trust in key opinion leaders (KOLs), and acceptance of the zero-COVID policy, were significantly correlated. Moreover, we conducted a Bonferroni correction to protect from Type I Error. Five correlation analyses on the same dependent variable would suggest a necessity for a new value of p equal to the original alpha-value (α_original_ = 0.05) divided by the number of correlations: (α_altered_ = 0.05/5) = 0.01. To decide if any of the five correlations is statistically significant, the *value of p* must be *p* < 0.01.

**Table 2:** Correlation among study variables.

	1	2	3	4	5
1. Collectivist issue interpretation	-				
2. Individualist issue interpretation	0.15^***^	-			
3. Trust in government	0.27^***^	−0.02	-		
4. Trust in key opinion leaders (KOLs)	0.18^***^	0.22^***^	0.52^***^	-	
5. Acceptance of the zero-COVID policy	0.13^***^	−0.25^***^	0.24^***^	0.01	-

Age, gender, education, monthly household income, political leaning, and number of children were controlled. *N* = 3,312. ^*^p < 0.05; ^**^p < 0.01; ^***^p < 0.001.

**Figure 2:**
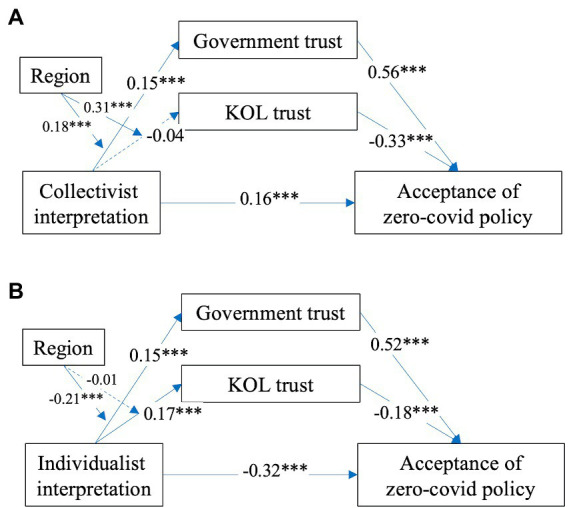
Test of the proposed model.

We then adopted PROCESS macro model 4 in SPSS (Hayes, 2017) to test the hypotheses. PROCESS is a widespread technique to study the mediation effects, and it is straightforward to use (Hayes, 2012). The results in Figure 2 showed that controlling for demographics, people’s collectivist issue interpretation of the pandemic was significantly correlated with their acceptance of the zero-COVID policy (β = 0.16, *p* < 0.001). Thus, *H1* was accepted. The zero-COVID strategy was more likely to be endorsed by people who regarded the COVID-19 epidemic as a collective concern. At the same time, the results revealed that people’s individualist issue interpretation of the pandemic was negatively correlated to their acceptance of the zero-COVID policy (β = −0.32, *p* < 0.001). Thus, *H2* was supported. In these two societies, the more that people tended to interpret the COVID-19 pandemic as an individual issue, the more likely they were to accept the zero-COVID policy.

In response to *H3*, which concerned the relationship between collectivist issue interpretation of the COVID-19 pandemic and trust in KOLs and the government, our results (Figure 2A) showed that collectivist issue interpretation was significantly correlated with trust in government (β = 0.15, *p* < 0.001). People were more likely to trust the government when they considered the COVID-19 pandemic a collective event. As for trust in key opinion leaders, our findings indicated that collectivist issue interpretation of the COVID-19 pandemic was not significantly correlated with people’s trust in KOLs (β = −0.04, *p* = 0.22). Thus, *H3* was partially supported.

Regarding *H4*, which focused on the relationship between people’s individualist issue interpretation of the COVID-19 pandemic and the trust in KOLs as well as the government, our findings (Figure 2B) demonstrated that individualist issue interpretation was positively correlated with trust in government in Mainland China and Taiwan (β = 0.15, *p* < 0.001). People who considered the COVID-19 outbreak a personal issue would show a higher level of trust in government. In terms of trust in key opinion leaders (KOLs), our results suggested that individualist issue interpretation of the COVID-19 epidemic was positively correlated to people’s trust in KOLs (β = 0.17, *p* < 0.001). Thus, *H4* was supported.

*H5* and *H6* implied the relationship between government trust, KOL trust, and people’s acceptance of the zero-COVID policy. Our findings (Figure 2) showed that trust in government positively correlated with people’s acceptance of the zero-COVID policy (collectivist model: β = 0.56, *p* < 0.001; individualist model: β = 0.52, *p* < 0.001). Thus, *H5* was supported. This indicated that those with higher trust in government were more likely to have positive attitudes toward the zero-COVID policy. Considering the KOLs trust, our results revealed that trust in KOLs negatively correlated with people’s acceptance of the zero-COVID policy (collectivist model: β = −0.33, *p* < 0.001; individualist model: β = −0.18, *p* < 0.001). People with a higher level of trust in KOLs would be less likely to adopt the zero-COVID policy. Thus, *H6* was supported.

Regarding *H7* and *H8*, which concerned the moderation effect of region on the relationships between people’s collectivist issue interpretation and their trust in the government and trust in KOLs. Our results (Figure 2A) demonstrated that region was a significant moderator of the relationship between people’s collectivist issue interpretation and their trust in the government (β = 0.18, *p* < 0.001). It also significantly moderated the relationship between people’s collectivist issue interpretation and trust in KOLs (β = 0.31, *p* < 0.001). Thus, *H7* was supported. In response to *H8*, which evaluated the moderating role of region, results (Figure 2B) revealed that region was a significant moderator of the relationship between people’s collectivist issue interpretation and their trust in the government (β = −0.21, *p* < 0.001). However, the region was found to be an insignificant moderator on the relationship between people’s individualist issue interpretation and trust in KOLs (β = −0.01, *p* = 0.70). Thus, *H8* was partially supported.

The results in Table 3 suggested the indirect effect of cultural values on the acceptance of the zero-COVID policy. For the path from collectivist issue interpretation of the COVID-19 pandemic to policy acceptance, trust in government was a significant mediator. Trust in KOLs was also a significant mediator of this path. For the path from individualist issue interpretation of the COVID-19 pandemic to policy acceptance, trust in government was not a significant mediator, while trust in KOLs was a significant mediator of the path. Moreover, the difference between two indirect paths (i.e., trust in government and trust in KOLs) was significant.

**Table 3:** Testing the indirect effect on acceptance of the zero-COVID policy.

Indirect Effect		Trust in government		Trust in KOLs	
		*B* (Boot SE)	Boot 95% CI	*B* (Boot SE)	Boot 95% CI
Collectivist model		0.164 (0.017)	[0.131, 0.199]	−0.059 (0.010)	[−0.080, −0.041]
Individualist model		−0.008 (0.007)	[−0.022, 0.006]	−0.029 (0.008)	[−0.044, −0.015]

Analyses conducted using PROCESS Model 4.

The results in Table 4 showed the conditional indirect effect of cultural values on zero-COVID policy acceptance. For the path from collectivist issue interpretation of COVID-19 epidemic to policy acceptance, trust in government was found to be a significant mediator in both conditions. Trust in KOLs was a significant mediator of this path in Mainland China but an insignificant mediator in Taiwan. For the path from individualist issue interpretation of COVID-19 pandemic to policy acceptance, trust in government was a significant mediator in both conditions. In both conditions, trust in KOLs was also a significant mediator of the path.

**Table 4:** Testing the conditional indirect effect on acceptance of the zero-COVID policy.

Indirect effect		Trust in government		Trust in KOLs	
		*B* (Boot SE)	Boot 95% CI	*B* (Boot SE)	Boot 95% CI
Collectivist model	Mainland (region = 1)	0.190 (0.019)	[0.154, 0.230]	−0.090 (0.014)	[−0.119, −0.064]
	Taiwan (region = 0)	0.087 (0.023)	[0.043, 0.134]	−0.012 (0.010)	[−0.008, 0.031]
Individualist model	Mainland (region = 1)	−0.028 (0.007)	[−0.042, −0.014]	−0.029 (0.008)	[−0.045, −0.014]
	Taiwan (region = 0)	0.080 (0.020)	[0.042, 0.120]	−0.031 (0.009)	[−0.050, −0.015]

Analyses conducted using PROCESS Model 7; trust in government and trust in KOLs were mediators; region was the moderator on the relationship between collectivist/individualist issue interpretation and mediators.

Generally, the direct and indirect effects of collectivist issue interpretation accounted for 11% of the variance in the zero-COVID policy acceptance. The direct and indirect effects of individualist issue interpretation accounted for 14% of the variance in the acceptance of the zero-COVID policy.

## Discussion

Under the continually severe pandemic, people’s attitudes toward the covid policy make a difference. The current study suggested that cultural values (i.e., collectivism vs. individualism) directly and indirectly influence people’s acceptance of the zero-COVID policy implemented by the government. With two constructs (i.e., trust in government and trust in key opinion leaders), this study unfolded the underlying mechanism toward the zero-COVID policy acceptance (i.e., efficacy mechanism).

By examining the theoretical framework in Figure 1, this study indicated the direct effect of cultural values on policy acceptance, the mediating role of trust in government, and trust in key opinion leaders in the direct relationship between two Chinese societies. Specifically, people who interpreted the pandemic as a collectivist issue were more likely to accept the zero-COVID policy, whereas individualist issue interpreters tended to reject the policy. Moreover, the cultural orientation of “I-C” both significantly and positively predicts people’s trust in government and KOLs. True distinctions lying in the contexts of two Chinese societies shape this relationship. Furthermore, trust in government promoted people’s policy acceptance, whereas trust in KOLs impeded their acceptance of the zero-COVID policy.

### Individualism–collectivism dichotomy and zero-COVID policy acceptance

This study suggested a direct relationship between cultural values and people’s acceptance of the zero-COVID policy in two Chinese societies. Specifically, collectivist issue interpretation positively predicted their policy acceptance. The more likely people interpreted the pandemic as a collectivist issue, the more likely they were to accept the zero-COVID policy. In addition, this study indicated a negative relationship between individualist issue interpretation and people’s acceptance of the zero-COVID policy in Mainland China and Taiwan. People who tended to interpret the pandemic as an individual issue were less likely to adopt the zero-COVID policy. This finding is congruent with previous studies concerning people’s cultural orientations in other contexts (e.g., Cheung and Chow, 1999). Ferle et al. (2008) suggested that respondents from Mainland China and Taiwan reported more collectivist values than individualistic values, demonstrating the proportional importance of collectivism in economies and advertising industries.

The individualism–collectivism dichotomy explained policy compliance behaviors among people in both societies. This finding suggested the significant impact of cultural values on individuals’ policy acceptance. Practically, governmental institutions may provide the collectivist framing about the pandemic that might enhance individuals’ social identity within a group (e.g., community and country), which would, in turn, cultivate a positive attitude toward the zero-COVID policy.

### Collectivist issue interpretation and higher levels of political trust

The current study revealed that collectivist issue interpretation would positively predict individuals’ trust in government in two Chinese societies. When people adopted a collectivist issue framing of the pandemic, they presented a higher level of trust in government. However, the relationship between collectivist issue interpretation and trust in KOLs was not significant.

The positive association between collectivist issue interpretation and trust in government is consistent with our expectation that given the self-efficacy mechanism, collectivists generally perceived less self-efficacy which impelled them to rely on and seek support from other entities (e.g., support from institutions or individuals; Choi and Kim, 2019). Moreover, this finding is in line with previous empirical studies that suggest collectivist values will enhance in-group trust (Pathak and Muralidharan, 2016) and that the country functions as a typical type of in-group (Yuki, 2003). Furthermore, the positive relationship between collectivist values and political trust has been revealed in studies conducted in other geographic regions (e.g., a 52-country study, Leonhardt et al., 2020; study in Australia, Shin and Park, 2004).

For the insignificant path from collectivist issue interpretation and trust in KOL, we conducted a *post-hoc* analysis to examine this relationship in the two societies, respectively. Results indicated that the collectivist issue interpretation of the COVID-19 pandemic positively correlated with people’s trust in KOLs in Mainland China (β = 0.27, *p* < 0.001) and presented a nonsignificant correlation in Taiwan (β = −0.02, *p* = 0.48). Possible explanations may lie in the nature of opinion leaders and the more diverse media environment in Taiwan. People with collectivist values were less likely to trust opinion leaders who were independent of the group since they regarded self-interest as a priority (Lukes, 1971). Also, opinion leaders in Taiwan are less likely to be affected by censorship and show a more pluralist inclination toward the zero-COVID policy, which leads to less alignment between them and collectivists.

### Individualist issue interpretation and higher levels of political/social trust

Consistent with our expectation, this study indicated a positive correlation between individualist issue interpretation and trust in key opinion leaders in two Chinese societies. When people adopted an individualist issue framing of the pandemic, they presented a higher level of trust in KOLs. This finding is consistent with previous studies revealing that individualists would autonomously present a higher level of social trust (Realo et al., 2008).

However, a positive association between individualist value and trust in government was revealed, which was inconsistent with our expectations and previous studies that suggested individualism to be the barrier to trust (Huang and Van de Vliert, 2006). We further conducted a *post-hoc* analysis to specify this relationship between two societies. Results indicated that individualist issue interpretation was negatively correlated with trust in government in Mainland China (β = −0.05, *p* < 0.001), which is congruent with the hypothesis. However, individualist issue interpretation was positively correlated with trust in government in Taiwan (β = 0.15, *p* < 0.001).

This may be due to the characteristics of individualism in Taiwan. Chiou (2001) indicated that vertical individualism was more prevalent in Taiwan compared to other regions (e.g., Argentina). Distinguished from other types of individualism, vertical individualism emphasized the perception of disparity from self and others (Singelis et al., 1995), with which people sought to be the best through competition (Triandis and Gelfand, 1998). Thus, they may pay close attention to institutions and individuals who offer information and opinions to improve themselves (Choi and Kim, 2019). Under the pandemic, it is plausible that people with a strong sense of vertical individualism in Taiwan recognized the crucial role of government and opinion leaders, sought support from them, and enhanced themselves to resist the virus.

### The moderating role of regions

Consistent with the hypotheses and the aforementioned *post hoc* analyses, this study suggested the differences lying in the two Chinese societies. Specifically, our findings indicated that the region positively moderated the relationship between people’s collectivist issue interpretation and their trust in the government and KOLs. Compared with people in Taiwan, the collectivist issue interpretation among people in Mainland China was more likely to influence their trust in entities.

This can be explained by the distinction of traditionalism and political systems in these two societies. Notably, Wong et al. (2011) have demonstrated the significant role of traditionalism in cultivating political trust among the public. Empirical studies have specifically compared Mainland China and Taiwan in terms of the influence of traditional culture on political trust (Shi, 2001), and discovered that in Taiwan, the performance of the government had a greater impact on political trust than traditional cultural values. Political trust in Mainland, on the other hand, was based on traditional cultural values, particularly the Chinese inclination toward hierarchical order and their collectivist identity (Shi, 2001). Hence, it is plausible to infer that the influence of collectivist issue interpretation on people’s trust in government and KOLs is greater in societies that praise traditional values than those do not. In addition, discrepancies in political systems may play a role in generating distinct political trust. Scholars have recognized that the political system may influence political trust in Chinese societies (Tang, 2005; Bjørnskov, 2007). Parallel to our results, previous studies have demonstrated a relatively stronger correlation between institutional confidence and trust in Mainland China compared with Taiwan (Steinhardt, 2012). China is a one-party state, while Taiwan is a young liberal democratic society. Therefore, it is reasonable to suppose that the effect of collectivist issue interpretation on people’s trust is more considerable in societies with a more authoritarian political system.

This study also demonstrated that region negatively moderated the relationship between people’s individualist issue interpretation and their trust in the government. Compared with people in Mainland China, the individualist issue interpretation among people in Taiwan was more likely to influence their trust in the government. For the individualist issue interpretation, it cannot be explained by the distinction of traditionalism in the two societies. A possible explanation lies in the difference in their strictness of preventive policies. Although both societies adopted the zero-COVID policy, policies in Mainland China were apparently stricter, with lockdowns and tightened controls in risky areas, than in Taiwan (South China Morning Post, 2022). People who interpreted the pandemic as an individualist issue were less likely to trust the government in societies with the very strict zero-COVID policy. Thus, the influence of individualist issue interpretation on people’s trust in government is greater in societies with less rigorous preventive policies during the COVID pandemic.

Overall, the region tends to be a contextual factor within the relationship among cultural values, trust, and policy acceptance. It includes various contextual indicators, such as cultural orientations, traditionalism, and the contemporary strictness of preventive policies in the regions. The influence of region depends on the joint effect of aforementioned indicators.

### Different roles of trust in government and KOLs on policy acceptance

The current study revealed trust in government to be a significant predictor of acceptance of the zero-COVID policy among people in both societies. When people present a higher level of governmental trust, a more positive attitude toward the zero-COVID policy will be cultivated. This finding is consistent with previous studies that suggest trust in government will promote policy acceptance (Leland et al., 2021). It sheds light on the idea that under the COVID pandemic, the government serves as the center of public attention, symbolizing its unity and power (Lee, 1977); people would support governmental actions due to their sense of patriotism. Practically, enhancing public trust in government is beneficial to promote the acceptance of the zero-COVID policy, especially for people who developed a strong sense of collective identity.

Additionally, trust in key opinion leaders was a negative predictor of people’s acceptance of the zero-COVID policy in both societies, which is also consistent with our expectations. It is also worth noting that the effect of KOLs is smaller than the effect of government in most of the models presented above. Therefore, it is safe for us to draw the conclusion that KOLs, though in vocal opposition to the zero-COVID policy, wield less power than the government in both societies.

Generally, this study contributes to the current studies of cultural values, trust, and policy acceptance in four ways: firstly, the study examines the relationship between cultural values and policy acceptance, while reiterating the effect of “I-C” dimension in people’s support for preventive measures under the pandemic. Secondly, the study presents an integrated model combining cultural values, trust, and policy acceptance. The model offers new insights to understand how people’s policy attitudes can be co-created by both cultural values and trust, generating new mechanisms to account for the formation of policy acceptance. Thirdly, the study presents differentiated impacts of social and political trust on the acceptance of zero-COVID policies and provides new implications for studying the relationship between trust and policy acceptance. Fourthly, by involving two Chinese societies (i.e., Mainland China and Taiwan), the study suggests the influence of contextual factors (e.g., traditionalism, strictness of preventive policies) within the whole process. Practically, rather than emphasizing the role of opinion leaders on media platforms, governmental institutions, and policymakers should prioritize their own ability and care for the public to promote government trust from citizens. They should endeavor to build social identity among citizens in order to cultivate their trust in government, which would eventually promote their acceptance of the zero-COVID policy implemented by the government.

### Limitations

The study has several limitations. First, in Mainland China, we used a non-probabilistic sample, although we attempted to enlarge the sample size and draw the sample based on the national parameters. Future studies may adopt probability sampling to replicate this study. Second, as a study that concerns the individualism–collectivism dichotomy, it is not sufficient to examine the framework in societies with a relatively higher level of collectivism. It should be noted that our research, despite exploring the cultural logic underlying the public support for zero-COVID policy, did not include other cultural factors apart from individualism–collectivism. Future studies should test a more integrated model in other societies. Third, in addition to mentioning the political impact on people’s policy compliance behaviors, future studies should investigate the effect of political systems. Fourth, given the differences between stringent control in Mainland China and the open policy of Taiwan, potential factors such as social norms and penalties for non-adherence to the norms may be analyzed in future studies. Finally, future studies should take into consideration possible confounders such as an individual’s experience of being infected during the pandemic.

## Data availability statement

The datasets presented in this article are not readily available due to privacy concerns. Requests to access the datasets should be directed to rhliu9-c@my.cityu.edu.hk.

## Ethics statement

The studies involving human participants were reviewed and approved by the Human Subjects Ethics Sub-Committee of City University of Hong Kong (Ethics approval reference nos.: 4-2020-05-F, 17-2021-23-F; Date of approval: 4 June 2020, 3 March 2021). The patients/participants provided their written informed consent to participate in this study.

## Author contributions

Y-HH, JL, and RL: conceptualization. RL and YL: methodology, software, and visualization. YL: validation and data curation. RL: formal analysis. Y-HH: resources, supervision, project administration, and funding acquisition. RL, JL, and YL: writing—original draft preparation and writing—review and editing. All authors contributed to the article and approved the submitted version.

## Funding

The work was supported by City University of Hong Kong under Grant (nos: 9380119, 7005703, and 9610573).

## Conflict of interest

The authors declare that the research was conducted in the absence of any commercial or financial relationships that could be construed as a potential conflict of interest.

## Publisher’s note

All claims expressed in this article are solely those of the authors and do not necessarily represent those of their affiliated organizations, or those of the publisher, the editors and the reviewers. Any product that may be evaluated in this article, or claim that may be made by its manufacturer, is not guaranteed or endorsed by the publisher.

## Supplementary material

The Supplementary material for this article can be found online at: https://www.frontiersin.org/articles/10.3389/fpsyg.2022.1047486/full#supplementary-material
