# Supplementary material for: Go for zero tolerance: Cultural values, trust, and acceptance of zero-COVID policy in two Chinese societies
Source: Front Psychol. 2022 Nov 22;13:1047486. doi: 10.3389/fpsyg.2022.1047486 (PMC9723468; doi:10.3389/fpsyg.2022.1047486)
Supplement: Supplementary file 1 [file Table_1.DOCX]

**Supplementary Materials**

| **Variables** | **Mainland China** | **Taiwan** |
| --- | --- | --- |
| ***Collectivist issue***  ***interpretation of pandemic*** | To what extent do you agree with the following statements about COVID-19?  1.This is mainly a social issue  2.This is mainly a group issue  3.This is mainly a national issue | |
| ***Individualist issue***  ***interpretation of pandemic*** | To what extent do you agree with the following statements about COVID-19?  1.This is mainly a privacy issue  2.This is mainly a personal issue | |
| ***Trust in key opinion***  ***leaders (KOLs)*** | How much do you trust the following institutions/people? | |
|  | 1.Experts in healthcare  2.Key opinion leaders (KOLs) on the WeChat official accounts and Weibo  3.TV & radio stations/newspaper editorials | 1.Experts in healthcare  2.Key opinion leaders (KOLs) on the Internet  3.TV & radio stations/newspaper editorials |
| ***Trust in government*** | How much do you trust the following institutions/people? | |
|  | 1.Central government  2.Local government  3.Centers for Disease Control and Prevention | 1.Central government  2.County/city government  3.Ministry of Health and Welfare  4.Centers for Disease Control and Prevention  5.County/City Public Health Bureau |
| ***Acceptance of the zero-covid policy*** | To what extent do you agree with the following statements regarding the Zero-covid Strategy?  1.I agree with the zero-covid/living with covid strategy  2.This zero-covid/living with covid strategy is effective  3.This zero-covid/living with covid strategy is beneficial  4.This zero-covid/living with covid strategy is wise | |
